# Effects of Stress Relaxation Aging with Electrical Pulses on Microstructures and Properties of 2219 Aluminum Alloy

**DOI:** 10.3390/ma9070538

**Published:** 2016-07-01

**Authors:** Jingsheng Tan, Lihua Zhan, Jiao Zhang, Zhan Yang, Ziyao Ma

**Affiliations:** 1 State Key Laboratory of High Performance Complex Manufacturing, Central South University, Changsha 410083, China; jingsheng@csu.edu.cn (J.T.); zhangjiao@dfl.com.cn (J.Z.); yangzhan@csu.edu.cn (Z.Y.); Maziyao@csu.edu.cn (Z.M.); 2 School of Mechanical and Electrical Engineering, Central South University, Changsha 410083, China

**Keywords:** 2219 aluminum alloy, stress relaxation aging, electrical pulses, mechanical properties, microstructure

## Abstract

To realize the high-efficiency and high-performance manufacture of complex high-web panels, this paper introduced electric pulse current (EPC) into the stress relaxation aging forming process of 2219 aluminum alloy and systematically studied the effects of EPC, stress, and aging time upon the microstructure and properties of 2219 aluminum alloy. It is discovered that: (a) EPC greatly enhanced the mechanical properties after stress relaxation aging and reduced the sensitivity of the yield strength for the initial stress under the aging system of 165 °C/11 h; (b) compared with general aging, stress relaxation aging instead delayed the aging process of 2219 aluminum alloy and greatly increased the peak strength value; (c) EPC accelerated the aging precipitation behavior of 2219 aluminum alloy and reduced transgranular and grain-boundary energy difference, thus leading to a more diffused distribution of the transgranular precipitated phase and the absence of a significant precipitation-free zone (PFZ) and grain-boundary stable phase in the grain boundary, further improving the mechanical properties of the alloy.

## 1. Introduction

To manufacture the complex panels of a rocket fuel tank, some developed countries have conducted many studies on creep/stress relaxation aging forming technology, i.e., a manufacturing technique in which the stress relaxation forming and aging heat treatment strengthening are carried out at the same time by using the creep/stress relaxation property and aging precipitation strengthening property of aldural [1,2]. The technology boasts possessing advantages such as even deformation, high forming accuracy, good repeatability, excellent forming efficiency, low residual stress, stable dimensions and uniform properties, and can achieve the spatio-temporal integration of the manufacturing environment centered by formation and heat treatment strengthening. For this reason, it could realize the collaborative formation and heat treatment strengthening manufacture of complex high-web panels to some extent [3,4]. A component’s stress state during the creep-loading, bending, and attaching-die process is rather complex, and its inside stress level is manifested as the distribution characteristic transitioning gradually from the maximum tension stress to the maximum compressive stress. Some studies have demonstrated that different stress levels will inevitably cause different creep aging precipitation processes at points inside the component and thus non-uniform component properties.

Besides the temperature and stress, electric pulse current (EPC) is also used to change the progress of the solid-state phase transformation. Many studies have confirmed that EPC can affect the phase transition process of the metal material. By applying a low-current-density direct electric current on the aging process of Cu-Cr-Zr alloy after solid solution and cold deformation, Wang et al. [5] concluded that the electric current exerts a great promotional effect on the process of precipitation. Huang [6] found that acting high-density EPC on copper-nickel alloy is capable of achieving rapid aging and the regular segregation of alloy elements. Liu et al. [7] pointed out that the high-density EPC treatment accelerates the growth rate of the γ’ phase, reduces the diffusion activation energy in the aging process and changes the distribution form ratio of the multiple precipitated phases. Wang [8] discovered that high-density EPC treatment can reduce the temperature of aging, improve the nucleation rate of carbides and change the distribution rule of precipitated phases in the grain boundary. Jia et al. [9] considered that EPC reduces the critical nucleation radius and nucleation energy. Conrad [10] held that the influence of the current on the solid-state phase transformation is due to the electromigration effect generated by the current. To [11] ascribed the promotional effect of the current on the phase change to the ponderomotive force difference generated by the current. Witt et al. [12] applied a DC current to Al-Cu interconnects, and found that the current’s effect can promote the precipitation of the Al_2_Cu phase.

The parameters frequently considered in the stress relaxation aging process are temperature, stress, and time, while the influence of EPC is rarely studied. Thus, the EPC is introduced into the stress relaxation aging process to influence the effect of the stress gradient on the precipitation and stress relaxation process in this paper. Due to its excellent properties in welding, corrosion and high-temperature mechanical properties, 2219 aluminum alloy has become the main material that is widely applied to fuel tanks of launch vehicles [13,14]. This paper took 2219 aluminum alloy as the subject of the research and introduced an EPC into the stress relaxation aging forming process of 2219 aluminum alloy to explore the influences of EPC, stress and aging time on the structure and properties of 2219 aluminum alloy, and thus offered a theoretical basis to manufacture high-efficiency and high-performance complex panel components of 2219 aluminum alloy.

## 2. Material and Methods

### 2.1. Material

**Figure 1:**
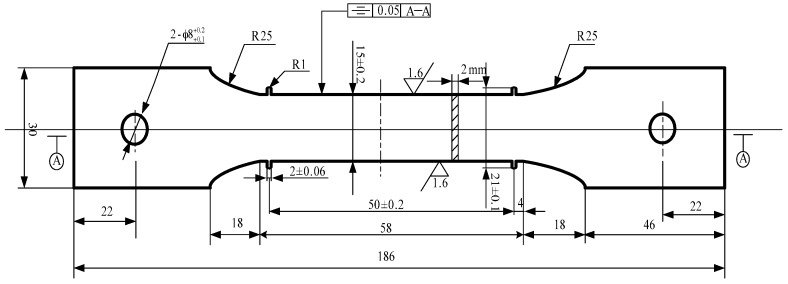
The size of the samples (unit: mm).

The material used here was 2219 aluminum alloy rolled plates with 7% pre-deformation, provided by the China Academy of Launch Vehicle Technology (Beijing, China). The chemical composition is shown in Table 1. Based on GB/T 2039-2012 [15], the plates were cut into 2-mm-thick standard creep specimens with Wire cut Electrical Discharge Machining (WEDM), as shown in Figure 1.

### 2.2. Methods

The stress relaxation aging test was carried out on an RMT-D10 electronic high-temperature creep rupture strength testing machine (produced by SUST, Zhuhai, China) with temperature control accuracy of ±2 °C and load accuracy of ±3 N. The pulsed power supply was switched on for electric stress relaxation aging experiment if the prepared sample was heated to aging temperature and loaded to target load. Temperature measurements were conducted at three positions of the upper, middle and lower part of the sample by thermocouple and the thermocouple at the middle part controlled the temperature during this experiment. The electric creep/stress relaxation experiment used the independently developed insulated fixture system, and the sample was insulated from the thermocouples using mica sheets.

A CMT-5504 electronic universal testing machine produced by SUST was used to conduct the tensile experiment for the sample with a tension speed of 2 mm/min. Three samples were tested for tensile properties and the average values were reported. A JEM-2100F field emission high-resolution transmission electron microscope (TEM) produced by JEOL (Tokyo, Japan) was employed to observe the microstructure of alloy samples. Before the observation, the sample was mechanically polished to 80–100 μm, shaped in a wafer of Ø 3 mm by the drilling machine, sprayed by the mixed liquor of 25%HNO_3_ + 75%CH_3_OH with the temperature of −25–35 °C, cooled down by the liquid nitrogen with the voltage of approximately 15 V, washed by ethyl alcohol for 2–3 min after being punched and produced into TEM observation sample.

## 3. Results and Analysis

### 3.1. Influence of the EPC on Properties of 2219 Aluminum Alloy after the Stress Relaxation Aging

Table 2 is the comparison of properties after stress relaxation with or without EPC under different initial stresses. The aging temperature and aging time are 165 °C and 11 h, respectively.

From Table 2, it can be shown that the yield strength presented the fluctuation tendency of an increase-decrease-increase as the stress increased; the tensile strength had a more hysteretic reaction on the initial stress than the yield strength and it was rather slightly affected by the initial stress (the difference between the maximum yield strength and tensile strength was 8.39 MPa/7.01 MPa, while the initial stress changed from 120 to 225 MPa). The initial stresses after the attaching-die of the components during the stress relaxation aging forming process are inconsistent, so the non-sensitivity of the mechanical properties to the initial stress after the stress relaxation is an essential condition of uniform component properties.

The EPC generally improved the mechanical properties of the 2219 aluminum alloy after stress relaxation under the same aging system of 165 °C/11 h (including yield strength, tensile strength and elongation), specifically to 3.3%, 3.3% and 3.6%, respectively, on average. Furthermore, it also reduced the sensitivity of the yield strength to the initial stress (the fluctuation was within 3.24 MPa).

### 3.2. Influence of Stress Relaxation Aging on the Evolution Rules for Properties of 2219 Aluminum Alloy

With the nucleation, growth and coarsening of the precipitated phase, the strength was observed to have a trend of reaching the peak before declining. Therefore, studies about the influences of aging time on the properties of 2219 aluminum alloy are necessary to find out the optimal aging time. Properties of the 2219 pre-deformation panel after stress relaxation formation are slightly affected by the initial stress (in Table 2), so 0 and 150 MPa were adopted in this experiment.

By setting the aging temperature at 165 °C, the constant-strain stress relaxation aging of 2219 aluminum alloy with an initial stress of 150 MPa and the mechanical properties under different durations of time with an artificial aging stress of 0 MPa are shown in Table 3.

From Table 3, it can be seen that the yield strength and tensile strength of 2219 aluminum alloy under the artificial aging of 0 MPa and constant-strain stress relaxation with an initial stress of 150 MPa presented the fluctuation tendency to increase before a decrease as the aging time was extended. The alloy strength reached its peak at 9 h after the artificial aging of 0 MPa, at which moment the yield strength and tensile strength were, respectively, 381.11 MPa and 457.25 MPa and the elongation was 8.46%. The alloy strength reached to its peak 15 h after constant-strain stress relaxation with an initial stress of 150 MPa, at which moment the yield strength and tensile strength were, respectively, 388.15 MPa and 472.15 MPa and the elongation was 10.98%. It is evident that the external stress with an initial stress of 150 MPa and a tendency of constant attenuation along with the time would delay the aging peak and aging process.

Cold deformation during the pre-deformation process produced a mass of dislocations inside alloy materials. On the one hand, the increased energy storage of alloy enhances the nuclear driving force of the precipitated phase, and on the other hand, the dislocation zone can gather solute atoms, segregate into Cottrell atmosphere, become a vantage point of nucleation, speed up the nucleation of the precipitated phase and accelerate the aging process. In regard to the introduction of external stress during the aging process, on the one hand, it generates slight but constant deformations in the alloy, continuously introduces dislocation in the alloy and thus accelerates the aging process [16]. On the other hand, the lattice structure of the alloy under the stress effect may vary slightly, and introducing extra the stress field may change the strain energy and exert an impact on the diffusion process [17]. However, there is a critical stress value in the aluminum alloy stress aging precipitation, and only when the external stress exceeds this critical stress value can it promote the precipitation of the atomic cluster and Guinier Preston (GP) zone [18]. Besides, the influences of external stress on the low-temperature aging precipitation are mainly exerted in the initial period [17].

Because the alloy material after the treatment of pre-deformation has already introduced a large number of dislocations and if the constant strain with an initial stress of 150 MPa is introduced as well at the moment, there would inevitably be certain dislocations generated by the small deformation which is triggered by the introduction of stress. Be that as it may, such dislocations may generate glide and climb motion under the co-activation of stress and temperature, due to which unlike dislocations can be canceled out and like dislocations can verge to the minimum energy state to lower the density of dislocations. At the same time, due to the gradual transformation into plastic deformation, the elastic deformation also contributes to the rapid decline and constant attenuation of external stress, resulting in the constant decrease of introducing the dislocation capacity and weakening the dislocation’s promotional effect on the aging precipitation. If the stress is attenuated below the threshold stress, the external stress may fail to promote the precipitation of the atomic cluster or GP zone. Hence, the constant-strain stress relaxation aging of 150 MPa lags behind the conventional artificial aging.

### 3.3. Influences of Electric Pulse on Evolvement Rules of Stress Relaxation Aging Properties of 2219 Aluminum Alloy

To explore the influences of electric pulse on evolvement rules of stress relaxation aging properties of 2219 aluminum alloy, setting the aging temperature as 165 °C and the initial stress as 150 MPa, this paper comparatively probed into the properties of aluminum alloy with or without electric pulse under different aging times, as shown in Table 4.

From Table 4, it can be seen that with the increase of the aging time, the yield strength and the tensile strength of the samples increase first and then decrease. EPC greatly accelerated the aging process of 2219 aluminum alloy, in which the yield strength and tensile strength 5 h after EPC aging were, respectively, 375.20 MPa and 458.56 MPa and the elongation was 13.02%. The mechanical property was obviously superior to that 11 h after conventional aging. The EPC stress relaxation aging reached its peak at 11 h, 4 h in advance of the conventional stress relaxation aging. At the same time, the EPC slowed down the property attenuation rate of the alloy after over-aging. In addition, when they reach the peak, the mechanical properties are the same in both cases.

### 3.4. Influences of Electric Pulse on Microstructure of 2219 Aluminum Alloy

**Figure 2:**
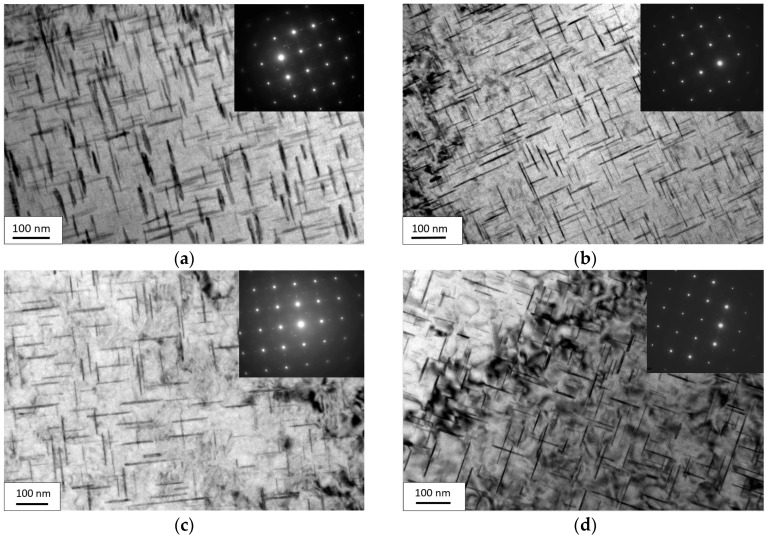
Transgranular TEM photo of 2219 aluminum alloy with or without EPC under the stress relaxation aging: (**a**,**c**) conventional stress relaxation aging of 150 and 210 MPa; (**b**,**d**) electric pulse stress relaxation aging of 150 and 210 MPa.

**Figure 3:**
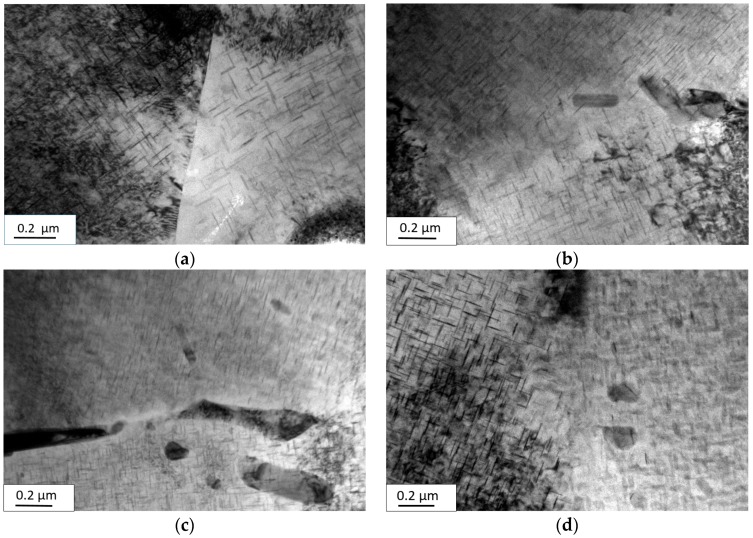
Grain-boundary TEM photo of 2219 aluminum alloy with or without EPC under stress relaxation aging: (**a**,**c**) conventional stress relaxation aging of 150 and 210 MPa; (**b**,**d**) electric pulse stress relaxation aging of 150 and 210 MPa.

The microstructures 11 h after aging with or without EPC under the aging temperature of 165 °C and different initial stresses (150 MPa and 210 MPa) are as shown in Figure 2 and Figure 3.

From Figure 2, it can be seen that under the temperature of 165 °C and 11 h after conventional stress relaxation aging, the sample with an initial stress of 150 MPa possessed a more intensive distribution of transgranular precipitated phases and a higher volume fraction of precipitated phases than those of the sample with an initial stress of 210 MPa; thus, it can be known that the sample with an initial stress of 150 MPa has a stronger strength of alloy based on the metal reinforcement theory [19]. However, there were more introduced dislocations at the early stage of stress when the initial stress was increased from 150 to 210 MPa, which reduced the difference between the transgranular energy and grain-boundary energy, making the transgranular and grain-boundary kinetics for precipitation more uniform and the precipitation-free zone (PFZ) at the grain boundaries more narrow, thus avoiding the continuous distribution state of the grain-boundary precipitated phases (Figure 3). All of these improved the mechanical properties of the alloy. Due to the competition between them, the yield strength with an initial stress of 210 MPa was much higher than that of 150 MPa and at the same time, the mechanical properties of 2219 aluminum alloy after conventional aging presented the small-range fluctuation state as the initial stress increased.

There was little difference in the distribution and volume fraction of transgranular and grain-boundary precipitated phases under 150 and 210 MPa after EPC stress relaxation aging. By comparing EPC aging and conventional aging, this paper found that transgranular precipitated phases had a more diffused distribution, more uniform dimensions and a higher volume fraction of precipitated phases (Figure 2). Since EPC promoted the movement of vacancy and solute atoms, solute atoms easily diffused and segregated to the transgranular dislocation and other nucleation sites. In this way, it may be easy for the nucleation of transgranular precipitated phases to form and grow and further weaken the advantage of nucleation in grain boundaries. Hence, there was no significant PFZ and stable phase in the grain boundaries (Figure 3), which can further improve the mechanical properties of the alloy.

## 4. Conclusions

1. The effects of initial stress on the mechanical properties of 2219 aluminum alloy stress relaxation aging are studied. It is found that stress relaxation aging is less affected by the initial stress, and the maximum yield strength/tensile strength difference is 8.39 MPa/7.01 MPa when the initial stress changes from 120 to 225 MPa.
2. EPC promotes the aging precipitation of 2219 aluminum alloy, significantly shortening the peak aging time. In addition, within the same aging time, the mechanical properties of the specimens with EPC stress relaxation aging are higher than that with conventional stress relaxation aging.
3. Compared with the conventional artificial aging, the external stress with an initial stress of 150 MPa and a tendency of constant attenuation over time delayed the aging process and slightly added to the peak strength value.
4. EPC accelerated the aging precipitation behavior of the 2219 aluminum alloy, reduced the transgranular and grain-boundary energy difference, contributed to a more diffused distribution of the transgranular precipitated phase, avoided obvious PFZ and stable phase at the grain boundaries, and thus further improved the mechanical properties of the alloy.

## Figures and Tables

**Table 1:** The main chemical composition of the 2219 aluminum alloy (wt %).

Element	Cu	Mg	Mn	Fe	Si	Zn	Zr	Al
Content (wt %)	5.8–6.8	0.02	0.2–0.4	0.3	0.2	0.1	0.1–0.25	Bal.

**Table 2:** Comparison of properties after stress relaxation with and without EPC under different initial stresses.

Initial Stress (MPa)	*σ_s_*/MPa		*σ_b_*/MPa		*δ*/%	
	Convention	EPC	Convention	EPC	Convention	EPC
120	376.09	385.12	460.17	465.97	10.70	9.94
150	372.80	388.38	454.29	476.85	9.02	9.41
180	372.62	387.95	456.97	473.41	8.78	8.90
195	370.23	386.35	459.96	474.77	9.07	10.09
210	377.63	386.96	461.30	474.92	8.90	10.21
225	378.62	388.36	458.17	476.85	8.57	8.47

**Table 3:** Mechanical properties of 2219 aluminum alloy at different aging times under the conditions of 165 °C and 0 MPa/150 MPa.

Aging Time (h)	*σ_s_*/MPa		*σ_b_*/MPa		*δ*/%	
	0 MPa	150 MPa	0 MPa	150 MPa	0 MPa	150 MPa
0	302.66	302.66	364.85	364.85	17.97	17.97
1	336.78	332.58	423.93	425.70	18.45	16.44
5	373.60	361.25	451.54	442.88	44.15	10.02
9	383.11	366.21	457.25	449.84	8.46	9.76
11	381.56	372.8	459.07	454.29	10.62	9.02
13	378.90	379.27	452.58	459.92	10.17	9.60
15	-	388.15	-	472.15	-	10.98
18	-	374.47	-	453.44	-	8.14

**Table 4:** Comparison of properties after stress relaxation aging with or without electric pulse under different aging times.

Aging Time (h)	*σ_s_*/MPa		*σ_b_*/MPa		*δ*/%	
	Convention	EPC	Convention	EPC	Convention	EPC
1	332.58	339.58	425.70	426.24	16.44	14.88
5	361.25	375.20	442.88	458.26	10.02	13.02
9	366.21	378.31	449.84	462.76	9.76	11.68
11	372.80	388.38	454.29	476.85	9.02	9.41
13	379.27	386.70	459.92	472.83	9.60	8.66
15	388.15	-	472.15	-	10.98	-
18	374.47	-	453.44	-	8.14	-
